# Flavonoids in prevention of diseases with respect to modulation of Ca-pump function

**DOI:** 10.2478/v10102-011-0019-5

**Published:** 2011-09

**Authors:** Lubica Horáková

**Affiliations:** Institute of Experimental Pharmacology & Toxicology, Slovak Academy of Sciences, SK-84104 Bratislava, Slovakia

**Keywords:** flavonoids, oxidative/nitrosative stress, Ca^2+^-ATPases in aging and diseases

## Abstract

Flavonoids, natural phenolic compounds, are known as agents with strong antioxidant properties. In many diseases associated with oxidative/nitrosative stress and aging they provide multiple biological health benefits. Ca^2+^-ATPases belong to the main calcium regulating proteins involved in the balance of calcium homeostasis, which is impaired in oxidative/nitrosative stress and related diseases or aging. The mechanisms of Ca^2+^-ATPases dysfunction are discussed, focusing on cystein oxidation and tyrosine nitration. Flavonoids act not only as antioxidants but are also able to bind directly to Ca^2+^-ATPases, thus changing their conformation, which results in modulation of enzyme activity.

Dysfunction of Ca^2+^-ATPases is summarized with respect to their posttranslational and conformational changes in diseases related to oxidative/nitrosative stress and aging. Ca^2+^-ATPases are discussed as a therapeutic tool and the possible role of flavonoids in this process is suggested.

## Introduction

Free oxygen and nitrogen reactive species (ROS, RNS) are participating in aging and age related diseases. The search for mechanisms of oxidative/nitrosative injury involved in diseases and aging is important for finding new therapeutic approaches. Impairment of calcium homeostasis is a consequence of oxidative/nitrosative stress. In recent years, many results were published showing that dysfunction of Ca^2+^-ATPases, the main calcium level regulating proteins, is included in the above mentioned conditions. In addition, modulation of Ca^2+^-ATPases by antioxidants may be a therapeutic tool for improvement of complications associated with human diseases and their experimental animal models. Flavonoids, in addition to their antioxidant properties exert beneficial effects based on their ability to bind to Ca^2+^-ATPases and thus to change their activity.

The importance of Ca^2+^-ATPases, their impairment and impact as a possible therapeutic tool in diseases related to oxidative/nitrosative stress has not yet been summarized. This review deals with the modulation of Ca^2+^-ATPases by oxidative/nitrosative stress *in vitro* and in diseases and with their potential therapeutic effect.

Flavonoids are phenolic compounds known as characteristic red, blue and purple anthocyanin pigments of plant tissues (Winkel-Shirley *et al.*, 2001). Flavonoids occur in food either as free monomers (quercetin, catechin) or oligomers (procyanidins), or they are bound to saccharides as glycosides. Consumption of flavonoid-rich food is associated with a lower incidence of coronary heart disease, myocardial infarction, cancer, neurodegenerative psychic diseases, and other chronic diseases. In the pathology of these diseases, oxidative stress has been assumed to play a role and flavonoids have been suggested to exert health benefits through antioxidant mechanisms. In addition to their antioxidant properties, flavonoids have been reported to exhibit other multiple biological effects, antiviral, antibacterial, anti-inflammatory, vasodilatory, anticancer, anti-ischemic, etc. (Procházková *et al.*, 2011). An important aim of the present review is to elucidate another mechanism by which flavonoids may exert health benefits, *i.e.* the modulation of calcium homeostasis and cell signaling *via* Ca^2+^-ATPase.

## Antioxidant properties and structure

**Figure 1:**
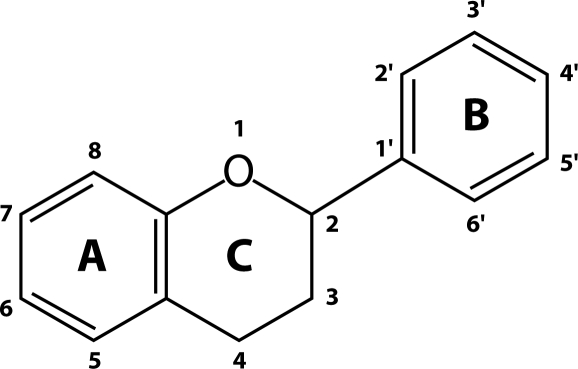
Basic structure of flavonoids.

Both the configuration and the total number of hydroxyl groups substancialy influence the mechanism of antioxidant activity (Heim *et al.*, 2002). The basic structure of flavonoids is depicted in Figure 1. The B ring hydroxyl configuration is the most significant determinant of ROS scavenging (Burda & Oleszek, 2001), whereas substitution of the rings Aand C has little influence on superoxide anion radical scavenging (Taubert *et al.*, 2003; Amic *et al.*, 2007). The *in vitro* antioxidant activity could be increased by polymerization of flavonoid monomers (proanthocyanidins known also as condensed tannins). The polymers of catechins are excellent *in vitro* antioxidants due to the high number of hydroxyl groups. The glycosylation of flavonoids reduces their *in vitro* antioxidant activity, compared with corresponding aglycones (Procházková *et al.*, 2011). Glycosylation of the 3-OH group has namely strong suppresive effect on their antioxidant activity (Rice-Evans *et al.*,^1996^).

**Figure 2:**
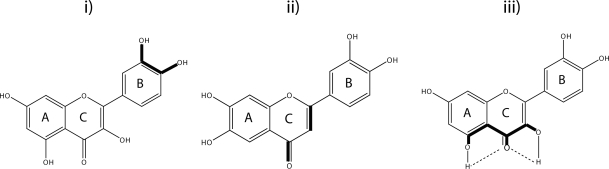
i) Ortho-dihydroxy (catechol)structure in the B ring, ii) 2,3-double bond in conjugation with 4-oxo function, iii) hydroxyl groups at positions 3 and 5.

Structural features of flavonoids required for efficient radical scavenging are i) an ortho-dihydroxy (catechol)structure in the B ring for electron delocalization, ii) 2,3-double bond in conjugation with a 4-oxo function in the C ring providing electron delocalization from the B ring, iii) hydroxyl groups at positions 3 and 5 providing hydrogen bonding to the oxo group (Croft 2006) (Figure 2).

## Ability to activate or inhibit enzymes

The ability to interact with various antioxidant enzymes belongs also to important mechanisms of flavonoids in preventing injury caused by free radicals. Protective effects of quercetin and catechin against hydrogen peroxide toxicity in cultured rat hepatocytes BL-9 were related to the activation of glutathione peroxidase (GPx) (Nagata *et al.*, 1999). Activation of survival signaling proteins (protein kinase B and extracellular regulated kinases) as well as increase of GPx and glutathione reductase (GR) caused by cocoa flavonoids was observed in human hepatocytes (Martin *et al.*, 2010). Luteonin induced human lung carcinoma cell apoptosis was accompanied by activation of antioxidant enzymes such assuperoxide dismutase (SOD) and catalase (CAT) (Leung *et al.*, 2006).

Flavonoids inhibit the enzymes responsible for superoxide production, *e.g.* xanthine oxidase and protein kinase C. Flavonoids have been shown to inhibit cyclooxygenase, lipoxygenase, microsomal succinoxidase and NADH oxidase. Flavonoids were found to exert an inhibitory effect on the expression of inducible nitric oxide synthase (NOS), yet they did not inhibit its activity. Several flavonoids, including quercetin, induce a reduction of ischemia-reperfusion injury by interfering with inducible NOS activity (Procházková *et al.*, 2011).

## Ability to modulate sarco/endoplasmic reticulum Ca^2+^-ATPase (SERCA)

### Physiological importance of SERCA

SERCA is an intracellular membrane-bound enzyme that utilizes the free energy of ATP to transport Ca^2+^ against aconcentration gradient. The physiological role of SERCA is to sequester cytosolic Ca^2+^ into membrane-bound intracellular compartments. SERCA-mediated Ca^2+^ uptake into stores plays a key role in maintenance of intracellular free Ca^2+^ levels within a physiological range. In addition, during contractions caused by elevated levels of intracellular Ca^2+^, accelerated sequestration of Ca^2+^ by SERCA mediates smooth, cardiac, and skeletal muscle relaxation. In non-contractile cells, such as proliferating smooth muscle cells or endothelium, SERCA regulates many cellular processes, including cell growth, apoptosis and migration. Ca^2+^-ATPase dysfunction has been detected in experimental and human pathology (Tong *et al.*, 2010; Inesi *et al.*, 2005.)

### Mechanisms of SERCA regulation

The mechanisms of intracellular calcium regulation by SERCA, involved in vascular relaxation, in cardiac and muscle relaxation, as well as in growth and differentiation, may include peroxinitrite (generated from nitric oxide and superoxide), which causes glutathione (GSH) to bind to SERCA cysteine thiols (Cohen *et al.*, 2006). GSH, when oxidized, is rapidly reduced and/or resynthetized to protect proteins from oxidation. With RNS GSH forms, S-nitroso glutathione (GSNO), sulfenic acid (GSOH), and GSH thiyl radicals (GS^**.**^), which can react with nearby protein thiols. Protein thiolate anions (R-S^**–**^) are predisposed to react with these species. Chemical adducts of protein thiols generated in the presence of RNS include Pr-SNO (generated by S-nitrosylation), Pr-SSG (generated by glutathiolation) and oxidized thiols (Pr-SOH), (Pr-SO_2_H), (PrSO_3_H), as well as intra- and inter-protein dithiols. All modifications, except those of sulfinic acid (Pr-SO_2_H) and sulfonic acid (PrSO_3_H), are reversible under physiologic conditions, enabling them to participate in cell signaling.

Another peroxynitrite product is nitrotyrosine. In SERCA2 in the aging heart and skeletal muscle nitration of vicinal tyrosines, Tyr 294 and Tyr295 was demonstrated on the luminal side of the membrane-spanning helix M4, correlating with partial inhibition of Ca-ATPase activity. This is suggesting a possible regulatory function in down-regulating mitochondrial energy production and the associated decrease in generation of reactive oxygen/nitrogen species (Bigelow, 2007). Nitrotyrosine is generated from nitric-oxide-derived oxidants, mainly of the CO_2_ adduct of peroxynitrite (ONOO^-^) or of NO_2_ (Pacher *et al.*, 2007). Peroxynitrite reacts rapidly with such nucleophiles as protein thiols and transition metal centers, and the physiologically abundant CO_2_, which occurs in millimolar concentrations in cells. The CO_2_ adduct nitrosoperoxycarbonate (ONOOCO_2_) rapidly homolyzes to ^•^CO^–^_3_ (carbonate radical) and.NO_2_ (nitrogen dioxide) to undergo aradical mechanism of tyrosine nitration. This involves hydrogen atom abstraction from the 3-position of tyrosine to form the tyrosyl radical that rapidly combines with.NO_2_ forming 3-nitrotyrosine. Protein nitration may also be formed by generation of ^•^NO_2_ by heme peroxidases, such asmyeloperoxidase and eosinophil peroxidase, in the presence of hydrogen peroxide.

Low concentrations of peroxynitrite induce S-glutathiolation of SERCA2 *in vivo*, whereas higher concentratioins of peroxynitrite result in irreversible modification of Cys^674^ to cystein sulfonic acid (Cys-SO_3_H) and accompanying nitrotyrosine modification (Cohen *et al.*, 2006).

### SERCA isomers and structure

Three genes for SERCA have been identified (SERCA1, SERCA2, and SERCA3), and different SERCA isoforms are expressed in tissue-specific and developmentally regulated patterns (Dremina *et al.*, 2007). The fast-twitch muscle isoform SERCA1 is restricted to fast-twich skeletal muscle, while SERCA2a is expressed in cardiac and slow-twitch skeletal muscle. The isoforms SERCA2b and SERCA3a-3f are present in alarge variety of tissues, including nonmuscle tissues.

SERCA belongs to the "P" family of cation transport ATPases, all of them forming phosphorylated enzyme intermediates by covalent interaction of the ATP terminal phosphate with an aspartyl residue at the catalytic site (Inesi *et al.*, 2005). ATPase includes amembrane-bound region connected through astalk to an extramembranous (*i.e.* cytosolic) headpiece. Ca-binding sites are located within the membrane-bound region, the ATP binding (nucleotide binding) site is situated in the cytosolic region of SERCA (Tupling *et al.*, 2001).

### SERCA, oxidation and natural plant products

SERCA pumps play a central role in maintaining low levels of free cytosolic calcium within cells. This Ca^2+^ pump is inhibited by a wide spectrum of hydrophobic molecules, a number of which are plant-derived natural products, such as thapsigargin (Wictome *et al.*,^1992^), curcumin (Bilmen *et al.*, 2001; Dyer *et al.*, 2002), and the flavonoid quercetin (Shoshan *et al.*, 1981). Flavonoids are able to bind to nucleotide binding sites, thus changing SERCA activity. Therefore one possible mechanism by which apoptosis is initiated by flavonoids is *via* Ca^2+^ pump inhibition leading to elevation of cytosolic [Ca^2+^], initiating Ca^2+^-dependent mitochondrial-mediated cell death (Ogunbayo*et al.*, 2008).

Natural plant extracts with antioxidant properties and prevention against pathological reduction of the Ca-pump may offer dual protection against some cardiovascular, skeletal muscle and inflammatory diseases. Recent findings showed that the inhibitory effect of HOCl, which reduced SR Ca^2+^-ATPase activity at least partially by structural changes, was prevented by standardized flavonoid extract from leaves of Ginkgo biloba (EGb761) (Štrosová *et al.*, 2005). It is known that flavonoids are able to bind to proteins, thus changing their structure and function (Rohdewald, 2002).

The standardized extracts of plant flavonoids, EGb761 and pycnogenol (Pyc) prevented sulfhydryl (SH) group oxidation and protein carbonyl (PCO) formation. Pyc in addition prevented thiobarbituric acid reactive species (TBARS) formation in SR oxidized by the Fenton system. The lower antioxidant effect of EGb761 compared with Pyc may be associated with the fact that OH-groups of compounds included in this extract and effective in scavenging free radicals are bound to glucose (Drieu, 1986). Mixtures of many compounds included in Pyc and EGb761 may have multiple and synergistic effects which could result in effective decrease of protein carbonyl formation. These extracts are also able to chelate Fe-ions and scavenge hydrogen peroxide and hydroxyl-radicals (Rohdewald, 2002; Sastre *et al.*, 1998). In spite of these antioxidant and scavenging effects exerted by EGb761 and Pyc, no protective effects on Ca^2+^-ATPase activity has been observed, on the contrary, an additional decreasing effect on the activity of this enzyme activity was observed. This effect may be caused by the interaction of flavonoids with free radicals derived from the Fenton system, inducing injury by this secondary reaction. In addition, Pyc is able to inhibit Ca^2+^-ATPase activity also in the absence of oxidants. It is able to bind to proteins, alter their structure and thereby modulate the activity of key enzymes (Packer *et al.*, 1999). The ability of Pyc to decrease Ca^2+^-ATPase activity under non-oxidized conditions was reported by Štrosova *et al.* (2006). Flavonoids included in the above mentioned standardized plant extracts may induce inhibition or stimulation of key enzymes (Rohdewald, 2002). EGb761 or its individual flavonoid compounds are able to bind to protein. Their binding to the nucleotide (ATP-binding) site of Ca^2+^-ATPase (Schroeter *et al.*, 2000) as well as interaction with ion channels was reported (Ishige *et al.*, 2001). We concluded that modulation of Ca-pump function by antioxidants depended on the mode of oxidative injury (Voss *et al.*, 2008). Both HOCl and radicals generated by the Fenton reaction are included in the processes of inflammation and are able to induce structural changes in proteins resulting in a decrease of Ca-pump function. The protective effect of trolox and EGb761 on SERCA activity may be based on the interaction of specific (HOCl related) secondary oxidative products with antioxidants. On the other hand, the same antioxidants, in spite of the fact that they protected SH groups and prevented TBARS or carbonyl generation, were found to be ineffective in preventing SERCA activity decrease induced by the Fenton reaction associated with HO and ferryl-radicals generation.

We conclude that the ability to protect Ca^2+^-ATPase activity by antioxidants depends on the mode of oxidative injury. We further suggest that conformational alterations in cytosolic nucleotide (ATP)-binding or transmembrane Ca^2+^-binding regions of Ca^2+^-ATPase, which are now under investigation in our laboratory, may be also critical for protective effects of antioxidants.

### Prooxidant properties of flavonoids

Under certain circumstances, flavonoids can act, as prooxidants, promoting the oxidation of other compounds. The prooxidant activity is probably directly proportional to the total number of hydroxyl groups in the flavonoid molecule (Cao *et al.*, 1997). Multiple hydroxyl groups, especially in the B-ring, significantly increased the production of hydroxyl radicals in the Fenton reaction, while on the other hand, mono- and dihydroflavonoids failed to exert any prooxidant activity (Heim *et al.*, 2002; Hanasaki *et al.*, 1994). There is also evidence that the 2,3-double bond and 4-oxo arrangement of flavones may promote the formation of ROS induced by divalent copper in the presence of oxygen (Sun *et al.*, 2010). Flavonoid prooxidant properties seem to be concentration dependent. An example may be rat liver microsomes, where quercetin and myricetin powerfully inhibited iron-induced lipid peroxidation at low micromolar concentrations (IC50≤1.5µM). However at 100 µM concentration, hydroxyl radical formation was greatly enhanced, up to eight-fold. We found that synthetically modulated derivatives of rutin exerted protective effects on ONOO^–^-mediated SERCA injury at low concentrations (5–50 µmol/l) and inhibitory at higher concentrations (100–250µmol/l), (Viskupicova *et al.*, 2009). Interestingly, the ability of rutin derivatives to inhibit SERCA activity in higher concentrations was not correlated with the prooxidant properties, probably due direct structural changes of SERCA by flavonoids.

Prooxidant or antioxidant properties of aparticular flavonoid depend most expressively on its concentration. The furher could be associated withcell signaling by which flavonoids contribute to the co-ordination of cell functions.

### Health benefits of green tea to humans

**Figure 3:**
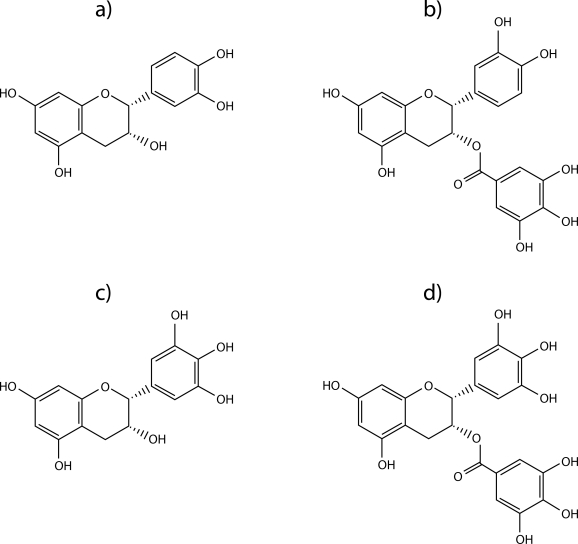
Chemical structure of (**a**) (–)epicatechin, (**b**) (–)epicatechin-3-gallate, (**c**) (–)epigallocatechin, (**d**) (–)epigallocatechin-3-gallate.

Tea is one of the most widely consumed beverages in the world. Polyphenolic compounds in green tea have recently received increased attention as preventive agents that may provide health benefits to humans. Green tea polyphenols include epigallocatechin-3-gallate (EGCG), epigallocatechin (EGC), epicatechin-3-gallate (ECG), and epicatechin (EC). The chemical structure of catechins is shown in Figure 3. The health benefits attributed to tea might result at least partly from the inhibitory effect of EGCG on Na^+^,K^+^-ATPase and Ca^2+^-ATPase activity (Ochiai *et al.*, 2009).

EGCG is the most abundant of these catechins (Bettuzzi *et al.*, 2006). It has been suggested to inhibit the activity of Na^+^,K^+^-ATPase in human erythrocyte ghosts (Rizvi & Zaid, 2005), P-type ATPases (gastric H^+^,K^+^-ATPase and sarcoplasmic reticulum Ca^2+^-ATPase). Daily intake of EGCG by drinking green tea may provide physiological benefits to humans *via* inhibition of Na^+^,K^+^-ATPase. EGCG and ECG exert their inhibitory effect on the membrane-bound Na^+^,K^+^-ATPase allosterically through interaction with plasma membrane phospholipid. The two galloyl-type catechins (EGCg and ECg) showed a stronger inhibitory effect on Na^+^,K^+^-ATPase than the other two catechins lacking galloyl groups (EGC and EC). EGCg and ECG have a greater affinity for membrane phospholipid than EGC and EC (Kamihara *et al.*, 2008).

## Cancer chemoprotection by flavonoids

Some flavonoids have been reported to reach levels of several micromolars in human blood plasma (Erlund *et al.*, 2002). Flavonoids are believed to be cancer chemoprotective due to their ability to sensitize some cells into undergoing apoptosis, a process that has become misregulated in many cancers. One hypothesis is that flavonoids are able to initiate apoptosis, especially in cancer cells, *via* a Ca^2+^-dependent mitochondrial pathway (Ogunbayo *et al.*, 2008). This pathway can be activated through an exaggerated elevation of cytosolic [Ca^2+^] and SERCA plays here an essential role by ameliorating such changes. Flavonoids (especially flavones) can inhibit the activity of Ca^2+^-ATPases isoforms SERCA1A and SERCA2B in the micromolar concentration range. Of the 25 flavonoids tested, 3,6-dihydroxyflavone (IC50, 4.6 µM) and 3,30,40,5,7-pentahydroxyflavone (quercetin) (IC50, 8.9 µM) were the most potent inhibitors. The polyhydroxylation of flavones is important for inhibition, with hydroxylation at position 3 (for SERCA1A) and position 6 (for SERCA2B) being particularly relevant (Ogunbayo *et al.*, 2008).

Natural flavonoid extract Pyc induced apoptosis in human mammary cancer cells, whereas normal mammary cells were not affected (Rodewald, 2002). Pyc is able to inhibit SERCA (Štrosová *et al.*, 2006; Voss *et al.*, 2008), which may be linked with its ability to induce apoptosis *via* an increase of tightly controlled cytosolic Ca^2+^. Similarly, other authors found an inhibiting effect of the anticancerogenic polyphenolic antioxidant curcumin against Ca^2+^-ATPase (Bilmen *et al.*, 2001). Interestingly, the apoptosis of peripheral blood lymphocytes was on the contrary significantly reduced in patients with systemic lupus erythematosus treated with Pyc (Rodewald, 2002).

In recent years, research has shown that some flavonoids can trigger apoptosis through the modulation of a number of key cellular signaling pathways that can cause increased cellular levels of caspases and decreased levels of antiapoptotic factors such as Bcl-2-type proteins (Ramos, 2007; Selvendiran *et al.*, 2006; Kuhar *et al.*, 2006).

Green tea has been suggestive to be a rather effective cancer preventive beverage. Suganuma (2011) studied the effects of combining EGCG and anticancer drugs, focusing on inhibition of cell growth and induction of apoptosis. Synergistic enhancement of apoptosis, gene expression, and anticancer effects were found on using various combinations of EGCG and anticancer drugs, including the combination of (-)-epicatechin (EC) and curcumin (Suganuma *et al.*, 2011).

Extracts of green tea and green tea polyphenols have exhibited inhibitory effects against the formation and development of tumors at different organ sites in animals (Yang & Wang, 2010). These include animal models for skin, lung, oral cavity, esophagus, stomach, intestine, colon, liver, pancreas, bladder, mammary gland, and prostate cancers. In addition to suppressing cell proliferation, promoting apoptosis, and modulating signaling transduction, green tea polyphenols, especially EGCG, also inhibit cell invasion, angiogenesis, and metastasis (Yang & Wang, 2010).

Flavonoids with inhibitory properties to Ca^2+^-ATPase activity may represent agents with the ability to affect cellular processes known to be regulated by Ca^2+^, including apoptosis, decreased synthesis of ATP and free radical formation.

### Green tea flavonoids and brain dementia

Green and black tea drinking (rich in a class of flavonoids named catechins) may help protect the aging brain and reduce the incidence of dementia like Alzheimer′s disease (AD) and Parkinson′s disease (PD). Neuroprotective/neuroregenerative effects of green tea catechins were found to act not only as antioxidant metal chelators but also as modulators of intracellular neuronal signaling and metabolism, of cell survival/death genes, and mitochondrial function. Thus, these dietary compounds are receiving significant attention as therapeutic multifunctional cytoprotective agents that simultaneously manipulate various brain targets (Mandel *et al.*, 2011).

## Cardioprotective effects of flavonoids

Higher flavonoid intake from fruits and vegetables is associated with decreased risk for the development of cardiovascular disease. The mechanisms explaining this observation remain unclear, but current evidence suggests that flavonoids may exert their effects through affecting cardiovascular risk factors. Flavonoids improve endothelial function, inhibit low-density lipoprotein oxidation, decrease blood pressure and improve dyslipidemia in studies focused on cocoa, soy, and green and black tea. Some polyphenols in their purified form, including resveratrol, berberine and naringenin, exerted beneficial effects on dyslipidemia in humans and/or animals. In a mouse model of cardiovascular disease, naringenin treatment, through correction of dyslipidemia, hyperinsulinemia and obesity, attenuated atherosclerosis. Thus the beneficial effects of flavonoids on multiple risk factors may explain, in part, their observed beneficial effects on cardiovascular disease (Mulvihill & Huff, 2010).

Several authors studied the effects of green and black tea or their individual components on the risk of cardiovascular diseases. Regular consumption of black and green tea may reduce the risk of cardiovascular disease. The cardiovascular health benefits of drinking tea are thought to be largely due to flavonoids. Isolated flavonoids found in tea have also been consistently shown to inhibit the development of atherosclerosis in animal models (Hodgson & Croft, 2010).

Molecular mechanisms of cardiovascular protection of green tea polyphenols, particularly EGCG, focusing on anti-oxidative and anti-inflammatory effects, were studied by Tipoe (2007). The most active component in green tea was found to be EGCG. Studies have shown that EGCG protects cellular damage by inhibiting DNA damage and oxidation of low density lipoproteins (LDL). One of the protective properties of EGCG is its ability to scavenge free radicals. EGCG was also able to reduce the inflammatory response associated with local tissue injuries, such as hepatocellular necrosis in acute liver injury induced by carbon tetrachloride. The protective effect of EGCG is due to its ability to decrease lipid peroxidation, oxidative stress and the production of nitric oxide (NO) radicals by inhibiting the expression of inducible nitric oxide synthase (iNOS). EGCG also ameliorates the overproduction of pro-inflammatory cytokines and mediators, reduces the activity of nuclear factor kappa-light-chain-enhancer of activated B cells (NF-kappa B) and transcription factor AP-1 and the subsequent formation of peroxynitrite with NO and reactive oxygen species. Thus EGCG effectively mitigated cellular damage by lowering the inflammatory reaction and reducing lipid peroxidation and NO-generated radicals leading to oxidative stress. Green tea is proposed to be a dietary supplement in the prevention of cardiovascular diseases, in which oxidative stress and proinflammation are the principal causes.

Other studies concerning flavonoid benefits were focused on cardiac hypertrophy. Cardiac hypertrophy is a pathological response of the heart to chronic pressure or volume overload. Cardiac hypertrophy is a risk factor for ischemic heart disease, arrhythmia, and sudden death. It is therefore important to determine the mechanism of the development of cardiac hypertrophy and prevent or treat it. Chronic low level of cardiac myocyte apoptosis was proposed to be a causal component in the pathogenesis of heart failure (Sheng *et al.*, 2007).

Many antioxidants have shown inhibitory effects on cardiac hypertrophy *in vitro* and *in vivo* induced by ROS (Sheng *et al.*, 2007; Tsujimoto, 2005). EGCG and green tea were found to exert protective effects against cardiovascular diseases (Chyu *et al.*, 2004; Townsend *et al.*, 2004). Green tea extracts and EGCG protected cardiomyocytes against ischemia/reperfusion-induced apoptotic cell death both *in vivo* and *in vitro* and effectively inhibited cardiac hypertrophy in mice and rats (Sheng *et al.*, 2007; Li *et al.*, 2006).

The inhibitory effects of EGCG on cardiac hypertrophy was tested by Sheng *et al.* (2007), and it was found that EGCG inhibited cardiac myocyte apopotosis and oxidative stress in pressure overload induced cardiac hypertrophy. EGCG also prevented cardiomyocyte apoptosis from oxidative stress *in vitro*.

Summarizing the above mentioned facts, we conclude that mechanisms of beneficial effects of flavonoids in cardiovascular diseases are based mainly on their antioxidant and antiinflammatory properties. On the other hand, the mechanism of cardiac hypethrophy prevention by flavonoids is suggested to operate *via* protection against apoptotic cell death of cardiomyocytes.

### Doxorubicin-induced cardiomyocyte injury

The effectiveness of the anticancer drug doxorubicin is severely limited by its cardiac side-effects related to cardiomyopathy and heart failure (Clement, 2009). The putative mechanism of doxorubicin-induced cardiotoxicity is the generation of reactive oxygen species during its intracellular metabolism (Choi *et al.*, 2007). An increasing number of studies suggest that disruption of calcium (Ca^2+^) homeostasis is another important cause for doxorubicin-mediated cardiotoxicity. Precise Ca^2+^ handling in cardiomyocytes is crucial for excitation–contraction coupling, the process that enables the heart to function effectively as a pump. Doxorubicin was reported to regulate the functions of L-type Ca^2+^ channels, Na^+^/Ca^2+^ exchanger, SERCA2 gene transcription and ryanodine receptors, thus modulating heart contractile function. (Zheng *et al.*, 2011)

Doxorubicin-induced cardiac myopathy was found to be reduced by antioxidant properties of EGCG. (Bast *et al.*, 2007). In addition, EGCG protection has also other aspects. One such aspect is its protein-binding ability which can help in the protection of cellular proteins against oxidative damage by reactive oxygen species (Hagerman *et al.*, 2003). As proteins are targets of oxidative damage, the interference of oxidative damage by EGCG may have apotential role in attenuating the development of cardiomyopathy.

Oxidative stress may also influence Ca^2+^ homeostasis by directly inducing mitochondrial permeability transition with modifications in mitochondrial calcium transport. The changes in calcium transport can further lead to tissue injury, cell killing and impaired cardiac contraction (Schimmel *et al.*, 2004). EGCG significantly increased cell viability and protected against apoptosis in doxorubicin-treated cells.

The effect of EGCG on Ca^2+^ handling was studied in cardiac myocytes and itwas found to protect myocytes against doxorubicin-induced intracellular calcium depletion (Zheng *et al.*, 2011).

In conclusion, EGCG-reduced cardiomyopathy after doxorubicin administration may be caused by several mechanisms: by antioxidant propeties of EGCG, its protein binding ability, protection against apoptosis of cardiac myocytes and protection against intracellular calcium depletion. In this respect, SERCA may be involved in the above mentioned protection, as flavonoids are able to bind to SERCA, change its activity and thus also the intracellular calcium level.

## SERCA dysfunction in diseases, posttranlational and conformational alterations

Conformational alterations of Ca^2+^-ATPases were studied in several diseases. Studies of ischemia/reperfusion in cardiac muscle and more recently in skeletal muscle showed that impaired sarcoplasmic reticulum (SR) function and Ca^2+^ homeostasis may be involved in the etiology of ischemia/reperfusion injury. Ischemia–induced structural changes in SR Ca^2+^-ATPase are associated with reduced enzyme activity in rat muscle. Experiments with prolonged ischemia in rat skeletal muscle revealed that structural modifications to SR Ca^2+^-ATPase can explain the alterations in Ca^2+^-ATPase activity that occur with ischemia (Tuplin *et al.*, 2001). They found that reduction in maximal SR Ca^2+^-ATPase activity in SR vesicles with ischemia was related to structural modification in the region of the nucleotide binding domain.

SERCA plays akey role in the relaxation of smooth, cardiac and skeletal muscle through the transport of cytosolic Ca^2+^ into SR or ER (MacLennan *et al.*, 1997; East, 2000; Sharov *et al.*, 2006). However also other physiological and pathological processes are associated with an abnormal activity and expression of SERCA, such as cell proliferation, apoptosis, Brody and Darier disease and cancer (Sharov *et al.*, 2006).

By reacting with superoxide anion, the less reactive NO forms the more thiol reactive oxidant peroxynitrite, which in turn can adduct glutathione (GSH) to SERCA cysteine thiols. The reaction of NO with superoxide anion is required, as indicated by the fact that mouse arteries in which superoxide dismutase is overexpressed relax less to NO (Adachi *et al.*, 2004). The majority of GSH on SERCA is bound to the most reactive thiol on cysteine-674, and mutation of this single cysteine prevents not only formation of most GSH adducts but also stimulation of Ca^2+^ uptake by NO. Tong *et al.* (2010) reported prevention of such stimulation of Ca^2+^ by Cys674 mutation associated with decrease of cell migration. NO function is impaired in a variety of cardiovascular diseases, including diabetic vascular disease and atherosclerosis, which are associated with irreversible oxidation of SERCA cysteine-674 (Tong *et al.*, 2010).

In atherosclerotic arteries, NO can not stimulate SERCA activity because of the irreversible oxidation of the cysteine-674 thiol caused by the high levels of oxidants accompanying the disease (Adachi *et al.*, 2004).

Viner *et al.* (1999a) reported inactivation of SERCA by higher, pathological levels of NO or peroxynitrite. Such inactivation can be physiologically detrimental as areduced function of SERCA has been associated with changed cellular response to apoptotic stimuli and also with an increased risk for cancer (Vanoverberghe *et al.*, 2004).

The increase of RNS in vascular diseases (such asatherosclerosis or diabetes) irreversibly oxidizes Cys674 or nitrate tyrosine residues at Tyr 296-Tyr297, which is associated with loss of SERCA function (Adachi *et al.*, 2010). Oxidative inactivation of target proteins for NO can be associated with the pathogenesis of cardiovascular diseases. Oxidative inactivation of SERCA is also implicated in dysregulation of smooth muscle migration, promotion of platelet aggregation and impairment of cardiac function, which can be associated with restenosis, pathological angiogenesis, thrombosis, as well as heart failure. Specific irreversible oxidative modifications consisting of sulfonylation at cysteine 674 and nitration at tyrosines 294/295 were found also in myocyte contractile dysfunction in the mouse heart mediated by reactive oxygen species (Lancel *et al.*, 2010).

NO-induced vasodilatation is impaired in patients with a variety of cardiovascular diseases, including diabetes, hypercholesterolemia (HC) and atherosclerosis (Adachi *et al.*, 2002, 2004; Williams *et al.*, 1996). SERCA 1 activity was markedly decreased in HC, although SERCA 2 protein expression did not change (Adachi *et al.*, 2001). The abnormal SERCA function in the HC rabbit aorta can be attributed to extensive oxidant-induced tyrosine nitration and thiol oxidation of SERCA (Adachi *et al.*, 2002; Ying *et al.*, 2008). Both tyrosine nitration and thiol oxidation may be attributed to excess production of peroxynitrite, the chemical product of NO and superoxide (Adachi *et al.*, 2002; Ying *et al.*, 2008).

In diabetic and insulin-treated diabetic Wistar rats, correlation was found between increase in tyrosine nitration of aortic SERCA2b and impaired aortic relaxation to acetylcholine, characteristic for the diabetic state (Adachi *et al.*, 2001). Prolonged treatment of the diabetic rat with insulin also impaired aortic relaxations that were attributed to dysfunction of the smooth muscle and were caused, at least in part, by SERCA dysfunction (Adachi *et al.*, 2001). In the streptozotocin-induced diabetic rat model, increased plasma angiotensin II and peroxynitrite levels impaired cyclic GMP-independent aortic relaxation, apparently by inhibiting SERCA (Taguchi *et al.*, 2007).

Additionally, the diabetic HC pig aorta contained SERCA2b in which the cysteine-674 thiol was oxidized, and this was prevented in pigs treated with insulin. Interestingly, the irreversible oxidation of SERCA in the diabetic pig aorta corresponded to a less intact 110 kDa SERCA protein and a lower molecular mass SERCA protein of approximately 70kDa. These studies suggest that SERCA degradation is associated with its oxidation and may play a role in progression of diabetic vascular disease (Ying *et al.*, 2008).

SERCA2 in platelets from patients with type 2 diabetes mellitus showed increased tyrosine nitration accompanied by inactivation of SERCA, elevated platelet free Ca^2+^ levels, and activated µ-calpain. The tyrosine nitration of SERCA2 and the activation of µ-calpain in platelets from healthy volunteers could be evoked by peroxynitrite *in vitro*, implicating the importance of oxidation of SERCA in diabetic patients (Randriamboavonjy *et al.*, 2008).

According to our recent results, development of adjuvant arthritis in an animal model was related to modulation of SERCA activity and correlated with oxidation of cysteine and nitration of tyrosine. We concluded that nitric oxide may regulate cytoplasmic Ca^2+^ level through conformational alterations in the transmembrane Ca^2+^ binding domain of SERCA (Strosova *et al.*, 2011).

Biological aging leads to oxidation and nitration of SERCA at Cys and Tyr residues (Restall *et al.*, 1986), accompanied by partial inactivation of SERCA activity and to a conformationally altered nucleotide binding site (Chen et al, 1999). Sharov *et al.* (2006) found that age-dependent loss of Cys residues may be partially responsible for the age-dependent decrease of the specific Ca-ATPase activity. Biological aging resulted in partial modification of Cys residues 377, 498, 525, 561, 614, 636, 674, 675, 774, 938. In the SERCA2a from aged slow-twich skeletal muscle (Viner *et al.*, 1999b) and heart (Knyushko *et al.*, 2005) accumulation of 3-nitrotyrosine (3-NT) at positions 294, 295, located at the lumen-membrane interface of the transmembrane helix M4, and at position 753 was found.

Summarizing SERCA dysfunction in the above mentionad diseases, it can be concluded that cysteine sulfhydryls, mainly in Cys 674 and tyrosine nitration usually at positions 294, 295, are the main posttranslational alterations of SERCA responsible for modulation of its activity, correlating with pathological disorders. Conformational alterations of SERCA in cytosolic nucleotide (ATP-binding) sites observed both in aging and in ischemia/reperfusion were found in cardiac and skeletal muscles, while conformational changes in the transmembrane domain of SERCA were associated with adjuvant arthritis. Several authors concluded that SERCA oxidative posttranslational modifications contributed to the pathophysiology of individual diseases and that preservation of SERCA function and/or increasing expression of SERCA may be a novel strategy against diseases associated with oxidative stress (Adachi *et al.*, 2010, Lancet *et al.*, 2010, Tong *et al.*, 2010, Inesi *et al.*, 2005).

## SERCA as a therapeutic target to prevent vascular disease development

Preventing SERCA oxidation and/or providing new SERCA protein may improve vascular disease development as indicated by several examples. In a study of impaired endothelium-dependent relaxations in the HC rabbit aorta, the antioxidant t-butylhydroxytoluene (BHT) dramatically improved the smooth muscle response to NO. The beneficial effects of the antioxidant depended on improvement of the response to NO in the smooth muscle cells (SMC) (Adachi *et al.*, 2002). BHT also restored the decreased aortic SERCA activity in HC and decreased tyrosine-nitrated SERCA without changing SERCA protein expression. These beneficial effects may depend on decreasing the direct effects of ROS/RNS on SERCA that are augmented in HC (Adachi *et al.*, 2002).

Another example is improvement of endothelium-dependent relaxation which precedes the regression of atherosclerosis caused by cholesterol lowering or antioxidant treatments. This suggests that improvement of the response to NO can limit the progression of atherosclerosis. The improvement in SMC function is associated with decreased oxidative modification of SERCA protein and improved Ca^2+^ uptake activity. This suggests that preservation of SERCA function may be regarded as a new target for the treatment of impaired vascular function.

In the streptozotocin-induced diabetic rat model, elevated plasma angiotensin II levels were improved by treatment with the angiotensin-converting enzyme inhibitor enalapril. As angiotensin II is a powerful inducer of NADPH oxidase, it is likely that enalapril helps prevent the diabetes-related impairment of SERCA function in the aorta by suppressing ROS formation (Taguchi *et al.* 2007).

The enhanced tyrosine nitration and inactivation of SERCA2 in platelets from type 2 diabetic patients were restored by treating the patients with rosiglitazone. This was accompanied by improvements in platelet [Ca^2+^]i and aggregation, indicating the importance of SERCA oxidation in platelet function in diabetic patients. Rosiglitazone also partially restored inhibition of platelet aggregation by NO, consistent with improvements in SERCA redox function (Randriamboavonjy *et al.*, 2008).

Nitrotyrosine, an indirect marker of peroxinitrite, was markedly increased in aortic smooth muscle from diabetic rats, while chronic enalapril administration reduced this increase. In streptozotocin-induced diabetic rats, angiotensin II production may lead to the generation of peroxynitrite and this may in turn trigger dysfunction of vascular smooth muscle SERCA (Taguchi *et al.*, 2007).

NO relaxes arteries, in part by stimulating Ca^2+^ uptake *via* SERCA in aortic smooth muscle, while HC impairs SERCA function and the response to NO. HC induces oxidative stress, which can impair SERCA function. Antioxidant BHT reversed impaired smooth muscle SERCA function in HC, which correlated with improved relaxation to NO (Adachi *et al.*, 2002).

## Conclusions

Both physiological and pathophysiological levels of ROS/RNS regulate cell functions by direct modifications of SERCA. This may contribute to the understanding of the way in which ROS/RNS regulate cell function and may also suggest new strategies to prevent vascular diseases and development of cancer.

Flavonoids are able to initiate apoptosis, especially in cancer cells, *via* a Ca^2+^-dependent mitochondrial pathway, which can be activated through an exaggerated elevation of cytosolic [Ca^2+^]_i_ and at least partially *via* sarcoplasmic/endoplasmic reticulum Ca^2+^-ATPases. Thus flavonoids may be auseful tool for preventing cancer development.

On the other hand, decrease of apoptosis is required in some cases. In cardiac hypertrophy *in vitro* and *in vivo* induced by ROS/RNS, the changes in calcium transport can further lead to tissue injury, cell killing and impaired cardiac contraction. Some flavonoids significantly increased cell viability and protected against cardiomyocyte apoptosis, *via* preservation of SERCA function, which can be a novel strategy against cardiovascular diseases associated with oxidative stress.
